# RNA polymerase mapping during stress responses reveals widespread nonproductive transcription in yeast

**DOI:** 10.1186/gb-2010-11-7-r75

**Published:** 2010-07-16

**Authors:** Tae Soo Kim, Chih Long Liu, Moran Yassour, John Holik, Nir Friedman, Stephen Buratowski, Oliver J Rando

**Affiliations:** 1 Department of Biological Chemistry and Molecular Pharmacology, Harvard University, 240 Longwood Avenue, Boston, MA 02115, USA; 2 Department of Biochemistry and Molecular Pharmacology, University of Massachusetts Medical School, 364 Plantation St, Worcester, MA 01605, USA; 3 School of Computer Science and Engineering, The Hebrew University, Givat Ram Campus, Jerusalem 91904, Israel; 4 The Broad Institute of Harvard and MIT, 7 Cambridge Center, Cambridge, MA 02142, USA; 5 The Alexander Silberman Institute of Life Science, The Hebrew University, Givat Ram Campus, Jerusalem 91904, Israel; 6 Current address: Division of Immunology and Rheumatology, Department of Medicine, Stanford School of Medicine, Stanford, CA 94305, USA

## Abstract

**Background:**

The use of genome-wide RNA abundance profiling by microarrays and deep sequencing has spurred a revolution in our understanding of transcriptional control. However, changes in mRNA abundance reflect the combined effect of changes in RNA production, processing, and degradation, and thus, mRNA levels provide an occluded view of transcriptional regulation.

**Results:**

To partially disentangle these issues, we carry out genome-wide RNA polymerase II (PolII) localization profiling in budding yeast in two different stress response time courses. While mRNA changes largely reflect changes in transcription, there remains a great deal of variation in mRNA levels that is not accounted for by changes in PolII abundance. We find that genes exhibiting 'excess' mRNA produced per PolII are enriched for those with overlapping cryptic transcripts, indicating a pervasive role for nonproductive or regulatory transcription in control of gene expression. Finally, we characterize changes in PolII localization when PolII is genetically inactivated using the *rpb1-1 *temperature-sensitive mutation. We find that PolII is lost from chromatin after roughly an hour at the restrictive temperature, and that there is a great deal of variability in the rate of PolII loss at different loci.

**Conclusions:**

Together, these results provide a global perspective on the relationship between PolII and mRNA production in budding yeast.

## Background

Gene transcription is one of the major mechanisms by which a cell responds to its environment, and the regulation of transcription has been one of the most intensively studied processes in biology over the past half century. In the past decade, the technical revolutions in whole-genome analysis have enabled unprecedented insights into the global changes in mRNA production in response to environmental cues, and into the roles for countless regulatory factors in the production of these mRNAs.

The abundance of mRNA in a cell is determined by the relative rates of production (transcription and processing) and destruction, integrated over time. Thus, while mRNA levels are easily measured using microarrays or deep sequencing, the correspondence between mRNA changes and transcriptional changes in response to a given perturbation is imperfect. This is widely understood, but the ease of mRNA measurements has led most genomic analyses of transcriptional regulation to use this readout rather than actual transcription rates.

A number of genome-wide studies have identified discrepancies between transcription rate *per se *and mRNA abundance. There is wide variation in mRNA half-life in budding yeast [1,2], from roughly 10 minutes to 50 minutes, and mRNA degradation is regulated in a condition-specific manner. In mammals, genome-wide analysis of ongoing transcription using nuclear run-ons or deep sequencing of small RNAs identified evidence for widespread nonproductive transcription by RNA polymerase II (PolII) [3-5]. Furthermore, global mapping of PolII localization in budding yeast revealed a large set of RNAs that were produced very 'efficiently', that is, where the mRNA level per polymerase was higher than the genomic average [6]. Finally, a great deal of recent literature has identified widespread instances of PolII 'pausing' at genes poised for rapid induction upon change in growth condition [7-14].

We therefore set out to explore the relationship between PolII levels and mRNA levels during response to environmental stimuli. We mapped PolII levels across the genome in budding yeast over a heat shock time course, and over a time course of exposure to the sulfhydryl-oxidizing agent diamide. In both cases, changes in mRNA levels were well-correlated with changes in PolII occupancy, and in general PolII changes typically explain approximately 50% of the variance in mRNA changes. We find evidence for widespread roles for several additional factors that cause deviations from expected mRNA abundance changes, including mRNA stability and nonproductive transcription. Specific types of genes are especially prone to nonproductive or regulatory transcription, such as genes involved in carbohydrate metabolism. Finally, we characterize the loss of PolII from chromatin over a time course of inactivation of the temperature-sensitive *rpb1-1 *allele of the Rpo21 subunit of RNA polymerase [15]. PolII stays associated with most genes for roughly an hour after shifting to the restrictive temperature, indicating that assays for ruling out transcriptional dependence of various nuclear processes should wait an hour after shifting this strain to the restrictive temperature.

## Results

We carried out genome-wide localization of PolII using an anti-Rpb3 monoclonal antibody as previously described [16,17]. Yeast were subjected to two distinct stress conditions that induce overlapping but distinct gene expression programs [18] - heat shock (to 37°C) and diamide treatment. PolII localization was measured by hybridization to genomic tiling arrays (60-bp probes every approximately 250 bp) at five time points (up to 2 hours) over each stress response time course (Additional file 1).

**Figure 1:**
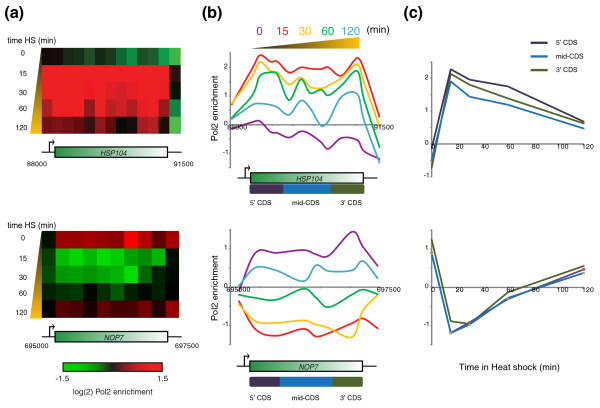
**PolII mapping during the stress response**. **(a) **Heat map view of *HSP104 *(top panel), an induced gene, or *NOP7 *(bottom panel), a repressed gene, during a time course of heat shock. **(b) **Data from (a), but plotted as a graph rather than a heatmap. Data on the y-axis are shown as log(2). Probes annotated as 5', mid, and 3' CDS are indicated below the gene annotation described (5' CDS = first 500 bp of coding sequence, 3' CDS = last 500 bp of coding sequence, mid-CDS = remaining coding sequence [16,19]). **(c) **Data from (a, b) were grouped by location as shown in (b). Averaged data for each group is plotted versus time at 37°C. HS, heat shock; Pol2, RNA polymerase II.

Broadly, our results capture expected aspects of the transcriptional response to stress in the budding yeast (examples shown in Figure 1). Data from both time courses were quite similar (R = 0.76 at t = 30, for example), consistent with the discovery that most of the expression changes in response to a given stressor correspond to a shared environmental stress response [18]. Dramatic gains or losses of PolII occurred at canonical stress-responsive genes: PolII levels increased dramatically (> 4-fold) over stress response genes such as *HSP104 *(Figure 1a-c, top panels) whereas PolII levels dropped precipitously (> 4-fold) over genes such as *NOP7*, involved in ribosome biogenesis (Figure 1a-c, bottom panels).

### Location of PolII along gene body

**Figure 2:**
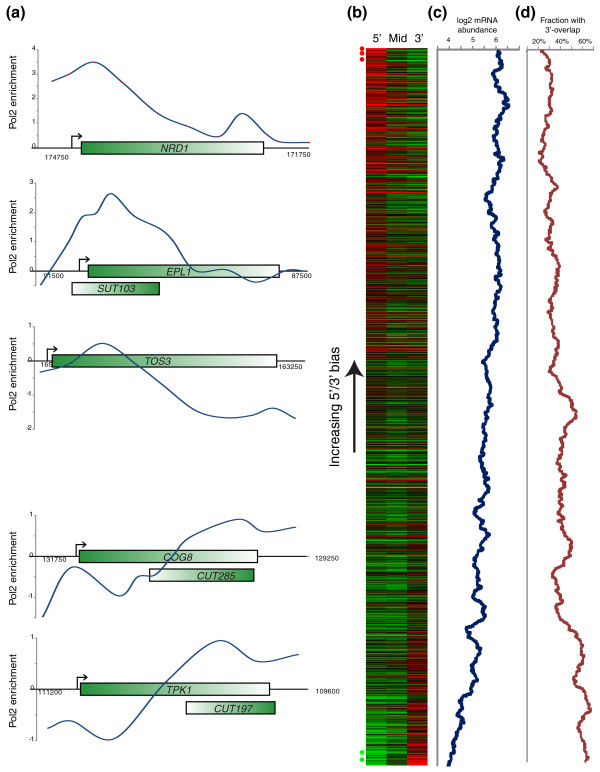
**Analysis of PolII location relative to ORFs**. **(a) **Example of genes with high (top three examples) or low (bottom two) ratios of PolII at the ORF 5' end relative to the 3' end. For each gene, PolII abundance is shown on the y-axis, and gene annotation and any cryptic transcripts from Xu *et al*. [22] are shown underneath. **(b) **All genes ordered by 5'/3' PolII ratio. For each gene long enough to have a mid-CDS annotation (that is, at least one microarray probe located > 500 bp from either end of the gene), the PolII enrichment at the 5' (first 500 bp), mid-CDS, and 3' (last 500 bp) are shown in the three indicated columns. **(c) **Genes with high 5'/3' PolII abundance are highly expressed. Log(2) of mRNA abundance data from Yassour *et al*. [21] is shown as an 80-gene running-window average, with genes ordered as in (b). **(d) **Genes with high 3'/5' PolII abundance are associated with overlapping transcripts. Genes were scored for 3' overlap with cryptic unstable transcripts (CUTs), stable unannotated transcripts (SUTs), or ORFs as annotated in [22], and a running window average is plotted ordered as in (b). Pol2, RNA polymerase II.

We next grouped data according to the location of the microarray probe within a given coding region, as previously described [16,19] - probes within the first 500 bp of a gene were annotated as 5' coding sequence (CDS), probes in the last 500 bp were annotated as 3' CDS, and any probes between these ends were annotated as mid-CDS (Figure 1c; Additional file 2). We noted a wide range in behaviors with respect to polymerase occupancy profiles over individual genes, with a spectrum ranging from high 5'/3' ratios to the converse (Figure 2a). As previously described [6,20], we found that several genes involved in transcriptional termination, such as *NRD1 *(Figure 2a) and *HRP1 *(not shown) exhibited high 5'/3' ratios of PolII. This is consistent with the described role for Nrd1 in feedback control of its own expression - when Nrd1 levels are adequate, transcription of the *NRD1 *gene undergoes premature termination, but when Nrd1 levels are low, termination becomes inefficient, leading to more transcription of full-length Nrd1 and restoration of high levels of the protein. Interestingly, other genes involved in transcriptional control also show exceptionally high 5'/3' ratios, including *EPL1 *(a NuA4 subunit) and *SMC2 *(a condensin subunit), suggesting that these genes may also be subject to regulation by transcriptional termination factors (Additional file 3).

Given the wide range of evidence for 'paused' RNA polymerase at the 5' ends of genes in flies, worms, mammals, and stationary phase yeast (see [7] for a review), we asked whether there was any evidence for paused PolII under our conditions. We therefore investigated what properties distinguish genes with high 5'/3' PolII ratios from those with the converse pattern. After selecting only those genes long enough to have a mid-CDS probe (that is, > 1 kb long), we sorted genes by the measured 5'/3' ratio of PolII in pre-stress midlog conditions (Figure 2b). Genes with relatively high 5' PolII tended to be expressed at higher levels [21] than genes with high 3' PolII (Figure 2c; Additional file 4). The high 5'/3' PolII ratios found at highly transcribed genes could indicate a rate-limiting transition from transcription initiation to elongation even at high transcription rates, which could result from PolII pausing or, alternatively, premature termination.

Conversely, we noted that many genes with high 3'/5' PolII ratios were associated with noncoding transcripts in mid-log growth conditions, either cryptic unstable transcripts (CUTs) or stable unannotated transcripts (SUTs) [22] (Figure 2a, bottom panels). This finding was general - a much higher fraction of genes with high 3'/5' levels of PolII exhibited overlap at their 3' ends with alternative transcripts than genes with high 5'/3' PolII ratios (Figure 2d). The high level of PolII at the 3' ends of these genes likely reflects transcription of the 3' CUT or SUT (our assay cannot distinguish the orientation of PolII movement); consistent with this idea, we found that genes with high levels of PolII at the 3' end of the gene exhibited high levels of the 'initiation' mark H3K4me3 at these 3' ends [19,23] (not shown). This transcription is nonproductive in the sense that the protein-coding RNA is not being produced by a significant fraction of polymerases occupying part of the gene body. Furthermore, the correlation between high 3'/5' PolII and low mRNA abundance suggests that overlapping transcription of 3' noncoding transcripts may play a more general role in control of productive transcription (see below).

**Figure 3:**
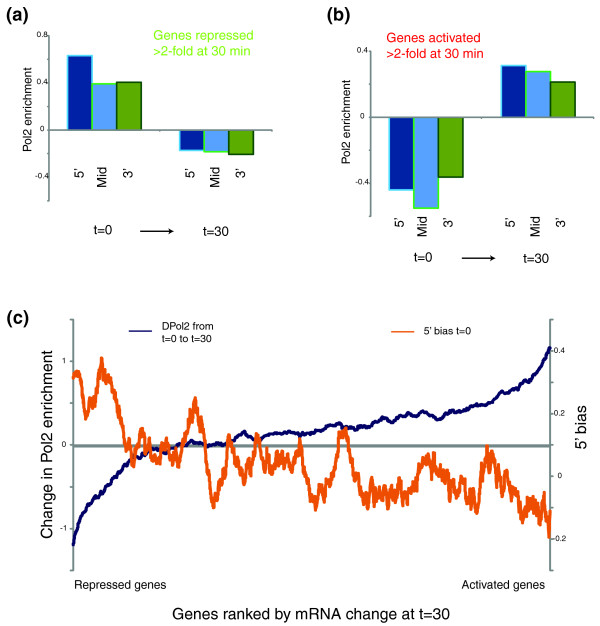
**Genes with 3'-biased PolII occupancy are preferentially activated during stress response**. **(a, b) **PolII occupancy at the 5' CDS, mid-CDS, and 3' CDS was calculated before (t = 0) and after (t = 30) heat shock for genes repressed (a), or activated (b) at least two-fold [18] during heat shock. **(c) **Genes are ordered by the level of induction after 30 minutes of heat shock. On the y-axis are plotted 80-gene running windows for change in PolII occupancy over mid-CDS, and for the 5'/3' PolII occupancy ratio at t = 0. DPol2 indicates change in PolII; Pol2, RNA polymerase II.

To explore how the localization of PolII along the gene body dynamically shifts during gene activation and repression, we calculated 5', mid, and 3' PolII abundance at all time points in the stress time courses. Genes were grouped by the extent to which their mRNA levels change at a given time point during the stress response, and 5', mid, and 3' CDS PolII enrichments were calculated for activated and repressed genes before and after 30 minutes of stress (Figure 3; Additional file 5). We see that changes in PolII levels generally correlate with changes in mRNA abundance, as expected. Furthermore, repressed genes shift from a pre-stress 5'-biased PolII distribution (characteristic of very highly expressed genes (Figure 2), which tend to be repressed during stress responses) to a flatter distribution after repression. Conversely, genes that are activated during stress initially exhibit slightly higher levels of PolII at the 3' end of the gene (Figure 3b), again suggesting that PolII is not paused at stress response genes in anticipation of stressors.

Interestingly, after activation of these genes, there is little 5' bias for PolII, on average, indicating that there is a subtle difference between highly expressed 'growth' genes and highly expressed 'stress' genes in terms of the kinetics or processivity of PolII transit over the gene. Many aspects of stress gene expression could cause this, such as distinct elongation factors traveling with PolII loaded onto TATA or non-TATA promoters. Alternatively, we favor a model based on trailing polymerases; stress genes in yeast exhibit 'bursts' of polymerases rather than the more evenly spaced polymerases seen at growth/housekeeping genes [24], and it has recently been shown that a trailing polymerase can aid the leading polymerase in overcoming the nucleosomal barrier to transcription [25,26], thus potentially allowing closely spaced polymerases to more easily overcome nucleosome-mediated delays.

### Transcriptional changes only partially account for mRNA abundance changes

To further investigate the relationship between mRNA abundance changes and transcriptional changes during stress, we grouped genes by k-means clustering of mRNA expression profiles over time in diamide (Additional file 6). These clusters correspond to various temporal profiles of gene activation/repression, including transient induction/repression, continuous induction/repression, and so forth. Broadly, the changes in PolII abundance at genes in each cluster mirrored the changes in mRNA abundance (Additional file 6b,c). Averaging mRNA changes and mid-CDS PolII changes shows nearly identical average profiles (Additional file 6d,e), indicating that, for example, genes exhibiting transient mRNA induction during diamide treatment were transiently transcribed, rather than continuously transcriptionally upregulated with subsequent regulation of mRNA stability contributing to the later decrease in mRNA abundance.

**Figure 4:**
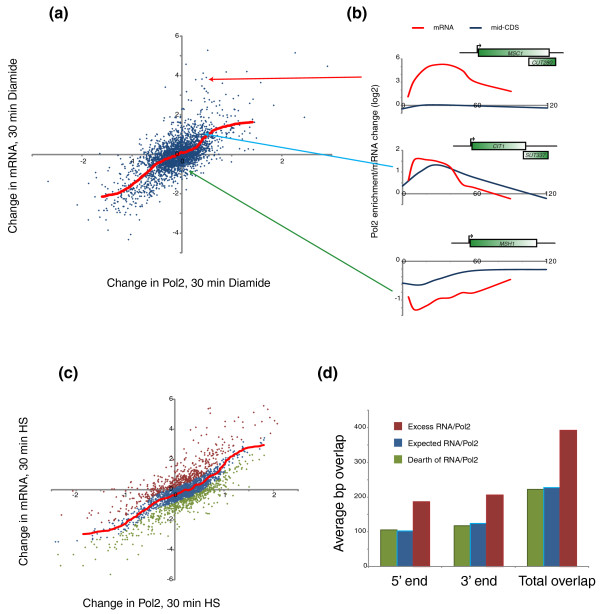
**Mismatches between mRNA production and changes in PolII occupancy**. **(a) **Scatterplot of mRNA change versus PolII occupancy change. PolII data are taken from mid-CDS probes, and change from 0 to 30 minutes of diamide treatment is shown on the x-axis. mRNA data from Gasch *et al*. [18] is shown on the y-axis. The red line shows the LOWESS fit of mRNA/PolII change. **(b) **Example genes with excess, typical, and a dearth of mRNA produced per change in PolII occupancy. Data for mRNA change and PolII change at mid-CDS are plotted at the same scale. **(c) **Definition of mRNA excess per PolII change. As in (b), but for 30 minutes of heat shock. Genes that fall more than 0.5 above (red) or below (green) the LOWESS fit (red line) of mRNA/PolII change are indicated. **(d) **Genes with excess mRNA production are subject to extensive overlapping noncoding transcription. Average extent of overlap with other transcripts defined in Xu *et al*. [22] are shown for the three gene classes defined in (c).

However, close examination of PolII changes within any given cluster reveal numerous examples where mRNA changes are not matched by PolII abundance changes. To investigate this phenomenon further, we compared the change in PolII abundance over mid-CDS probes and the corresponding change in mRNA abundance at varying times after induction of the stress response (Figure 4a,c; Additional file 7). We observed the expected positive correlation between PolII changes and changes in mRNA abundance, but there was significant variation as well - changes in PolII abundance typically accounted for approximately 50% of variance in mRNA abundance in this analysis. Examples of genes exhibiting high or low mRNA production per change in PolII occupancy are shown in Figure 4b.

To quantify the variability in mRNA produced per PolII molecule, we first calculated the average mRNA change per change in PolII as a LOWESS fit (red line in Figure 4a,c). Deviation from the typical mRNA change per PolII change was then defined as mRNA 'excess' or 'dearth' - genes that fall well above the red line in Figure 4a,c correspond to genes where the change in mRNA abundance measured in heat shock or diamide is significantly greater than the change in mRNA for most genes with the same change in PolII abundance. Examples of genes exhibiting high levels of mRNA excess or dearth are shown in Figure 4b. Genes exhibiting a relative excess of mRNA produced per PolII change were enriched in a variety of related Gene Ontology categories, such as 'hexose metabolic process' (*P *< 1.50e-13), and 'carbohydrate metabolic process' (*P *< 6.02e-11) (Additional file 8), as well as several relatively nonspecific Gene Ontology terms (see Discussion). Genes producing a relative dearth of mRNA per PolII change were enriched for Gene Ontology categories such as 'cell cycle' (*P *< 5.42e-9), and 'ncRNA metabolic process' (*P *< 3.38e-6).

Interestingly, genes for which excess mRNA was produced per PolII change often were associated with overlapping noncoding mid-log-expressed transcripts as defined by Xu *et al*. [22]. We found that this phenomenon was general, with a much greater extent of ORF overlap (*P *= 1.33e-5) with other transcripts at genes producing excess mRNA/PolII (Figure 4d; Additional file 9). This result suggests that in mid-log growth, much of the PolII occupying these genes is engaged in nonproductive transcription. Upon stress, we speculate that this nonproductive or regulatory transcription is repressed, allowing a greater fraction of productive PolII molecules to transcribe the coding region (Additional file 10). Consistent with this idea, we found that genes exhibiting excess mRNA production after treatment also tended to be associated with high 3'/5' PolII levels during mid-log growth (Additional file 11). Conversely, we speculate that genes exhibiting a dearth of RNA produced per change in PolII might be subject to an increased level of nonproductive transcription under stress, but since prior transcript mapping studies have not touched on heat shock or diamide conditions, these putative CUTs and SUTs have yet to be identified.

Because we used data from another lab's study for mRNA levels, we carried out our own measurements of mRNA changes at 30 minutes of heat shock (from the same culture used for PolII chromatin immunoprecipitation (ChIP)) using an oligonucleotide microarray, and repeated the analyses of Figure 4c,d. Our mRNA data were well-correlated with that of Gasch *et al*. [18] albeit with reduced dynamic range (Additional file 12a). Importantly, we reproduced the discovery that genes exhibiting 'excess' mRNA produced per PolII were associated with greater overlaps with CUTs and SUTs (Additional file 12b,c), validating the conclusions drawn using another lab's mRNA dataset.

### Characterization of the rpb1-1 allele

**Figure 5:**
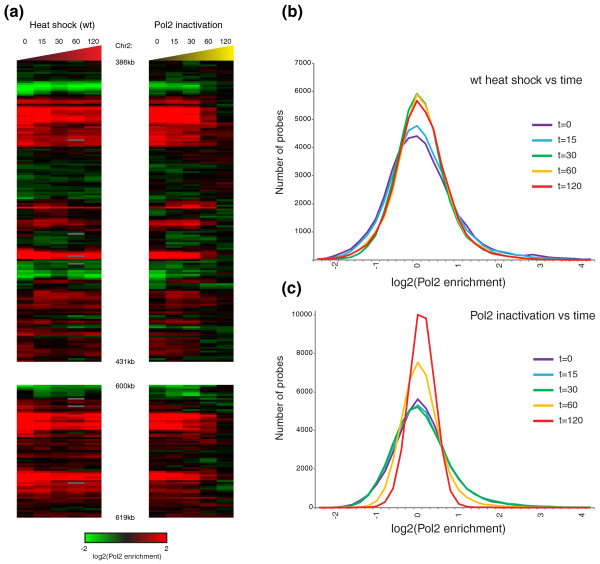
**Analysis of PolII occupancy in the *rpb1-1 *mutant**. **(a) **Examples of time course data from wild type (wt; left panels) or *rpb1-1 *yeast (right panels) during heat shock time courses. Chromosome coordinates are indicated between the two sets of panels. Note the decrease in dynamic range in the right panels in the last two columns, manifest as decreased color saturation in the rightmost two columns. **(b, c) **Histograms of microarray probe values for wild-type (b) or *rpb1-1 *(c) cells at varying times. Narrowing of the histogram in (c) indicates loss of PolII enrichment after approximately 1 hour of treatment with the restrictive temperature.

The *rpb1-1 *[15] temperature-sensitive allele of the gene encoding the major PolII subunit Rpo21 (or Rpb1) is widely used to establish whether a given change in some cellular behavior (such as chromatin structure) is transcription-dependent. We therefore sought to fully characterize the behavior of PolII along the genome upon shift to the restrictive temperature. We carried out PolII ChIP as above, in this case shifting *rpb1-1 *yeast from 24°C to 37°C for the same time points used for the stress time courses (Figure 5a).

At early time points (up to 30 minutes), PolII occupancy patterns were similar in wild-type and *rpb1-1 *yeast - PolII was recruited to *HSP104 *at early heat shock time points, for example (not shown). However, at 1 and 2 hours post-shift, we observed a dramatic decrease in the dynamic range of PolII abundance over the genome (Figure 5). Since microarrays are normalized to an average log2 enrichment of zero, this loss of dynamic range is the expected behavior if PolII association with the genome was globally diminished at these time points. This finding indicates that PolII is still associated with the genome 30 minutes after shifting *rpb1-1 *yeast to the restrictive temperature - extensive PolII dissociation from the genome does not occur until between 30 minutes and 1 hour after temperature shift, and is by no means complete even after 1 hour. Consistent with this, a prior study also found continued PolII association with the genome 45 minutes after inactivating the *rpb1-1 *mutant [27].

Is PolII loss uniform across the genome? There is some correlation evident between PolII abundance before and after PolII inactivation - loci that are highly enriched with PolII at the permissive temperature generally are associated with more PolII at 2 hours than are probes that are initially depleted of PolII (Additional file 13). Do some types of genomic loci maintain PolII more than others? For each of several types of genomic loci [16,19], we aligned loci by initial PolII abundance and plotted a running window average of PolII abundance after 2 hours at 37°C (Additional file 13a). This analysis reveals that PolII is maintained at the middle and 3' ends of genes to a greater extent than at the 5' end, suggesting that some polymerases may be capable of finishing a round of transcription prior to dissociation from the genome. Alternatively, it is possible that PolII located at the 3' ends of genes is somehow protected from dissociation.

Finally, we asked whether PolII could be recruited to the genome in *rpb1-1 *yeast at the restrictive temperature. *rpb1-1 *yeast were shifted to 37°C for 10 minutes, then subjected to diamide stress for 15, 30, or 60 minutes while maintaining the restrictive temperature. While heat shock and diamide both induce a common stress response, diamide also induces transcription of a specific set of genes that do not respond to heat shock [18], providing test loci to determine whether diamide-specific transcriptional changes are possible after 10 minutes of PolII inactivation.

**Figure 6:**
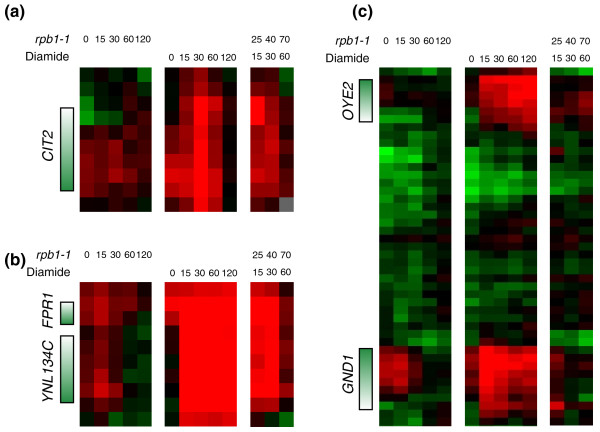
**PolII can still be recruited even after shifting *rpb1-1 *to 37°C**. **(a-c) **Examples of time course data from cells shifted to 37°C (left panel), treated with diamide (middle panel), or shifted to 37°C for 10 minutes before diamide addition (right panel). Panels (a, b) show regions where PolII is recruited to diamide-specific genes despite being at the *rpb1-1 *restrictive temperature, whereas (c) shows diamide-specific genes that fail to recruit PolII.

Surprisingly, PolII was recruited to a subset of diamide-specific genes under these conditions (Figure 6), indicating that not only can PolII maintain contact with the genome under these conditions, but it can still be recruited. PolII occupancy over some of these genes was not restricted to the promoter, suggesting that it might even transit the ORF under these conditions. Interestingly, only a subset of diamide-specific genes were capable of recruiting PolII after 10 minutes at the restrictive temperature. The difference between these two sets of diamide-specific genes is not apparent to us at present.

## Discussion

Here, we report dynamic whole-genome mapping of PolII occupancy during several different stress time courses. Our major findings are: 1, transcriptional changes in response to stress are only partly reflected in mRNA abundance; 2, widespread cryptic transcription likely contributes to gene regulation during stress response; and 3, some PolII maintains contact with the genome, and is even recruited, well after mRNA synthesis is thought to have stopped in the *rpb1-1 *mutant.

Most interestingly, we find widespread mismatches between changes in PolII and changes in mRNA abundance during two stress response time courses. While PolII recruitment to a given gene is correlated with an increase in expression of that gene, the quantitative level of mRNA change for a given relative change in PolII recruitment is highly variable. A number of factors could explain variability in mRNA production per PolII, such as regulated mRNA stability [2]. Indeed, we find that genes with excess mRNA production tended to exhibit longer half-lives (Additional file 14). However, genome-wide mRNA half-lives have typically been measured in the *rpb1-1 *strain, which appears to upregulate stress genes for at least some time during the shift to the restrictive temperature [1], indicating that the long mRNA half-lives for stress-related transcripts likely include effects of increased mRNA production during these time courses.

Here, we additionally find that genes exhibiting unusually high levels of mRNA produced per change in PolII generally exhibit greater overlap with cryptic and stable noncoding/unannotated transcripts (Figure 4d; Additional file 12c). Furthermore, we note that many poorly expressed mRNAs are associated with overlapping transcripts before stress (Figure 2d). Together, these observations support a model where some of the PolII associated with such a gene in mid-log growth is engaged in nonproductive (in some cases regulatory) transcription [22,28-35] (Additional file 10). Upon stress, upregulation of the ORF promoter, downregulation of the CUT/SUT promoter, or both, would result in a higher proportion of PolII molecules associated with a gene being engaged in productive transcription. We suspect that each of these three possibilities occurs at different genes.

We further speculate, then, that mismatches in which less mRNA is produced per PolII change represent genes where nonproductive transcription is induced in stress (see, for example, altered SUT expression in stress in [36]). As genome-wide datasets that identified CUTs and SUTs in budding yeast were not derived under stress conditions, these putative stress-specific transcripts would not have been identified in prior studies.

Interestingly, we found that genes involved in carbohydrate metabolism as a class are more subject to excess mRNA production than other gene sets (Additional file 8). Previous studies of cryptic unstable transcripts found that genes involved in glucose metabolism were significantly enriched for sense CUTs [37], consistent with our finding that genes exhibiting excess mRNA production were associated with overlapping CUTs or SUTs (Figure 4d). What is the biological rationale for regulation of carbohydrate-related genes by overlapping transcription? In the cases of nucleotide metabolism and termination factors [6,37], regulation by CUTs appears to provide a mechanism for feedback regulation of the relevant genes. In the case of carbohydrate metabolism the basis for direct feedback is less clear, although given the widespread mechanisms by which a cell's metabolic state can influence chromatin regulators' activities [38], we speculate that control of CUT transcription or termination could globally respond to NAD/NADH ratios or some other aspects of global cellular metabolism.

Finally, we extensively characterize the widely used temperature-sensitive *rpb1-1 *mutant in PolII. Many or most published studies use 15 minutes of inactivation of this allele to address the role of transcription in a given process (nuclear pore association, nucleosome positioning, and so on). However, here we find that PolII remains associated with the genome for approximately an hour before dissociating. Furthermore, we find that after 10 minutes in restrictive temperature PolII can still be recruited to newly activated genes. Prior studies with this mutant have shown a decrease in mRNA production [15] and in permanganate sensitivity [39] after 15 minutes of heat inactivation of this mutant, while our results show continued genomic association of PolII with the genome for at least another 15 minutes after this time. These different assays suggest that inactivating this mutant results first in loss of productive transcription without concomitant dissociation from the genome, followed after some time by dissociation from DNA. Thus, these experiments indicate that care must be used when interpreting the results of experiments with this mutant, and that longer incubation at restrictive temperature is required before PolII disengages from the genome.

Together, our results provide a broad perspective on the relationship between PolII and gene expression. These results have particular importance for studies attempting to use genomic sequence to understand transcriptional regulation - while the role of promoter sequence in the regulation of transcription is of course a major factor in the transcriptome, a great deal of variability in mRNA abundance may result from upstream or downstream regulatory promoters. Future computational studies will no doubt need to take local genomic structure-mediated effects such as these into account [40] in order to achieve a quantitative predictive understanding of how gene regulation derives from genomic sequence.

## Conclusions

Our results emphasize the ubiquity and plasticity of nonproductive transcription in budding yeast. Quantitative models of transcriptional regulation will be better served by focusing on PolII than on RNA abundance measures, as RNA abundance reflects a multitude of regulated processes from production to degradation. Finally, results from experiments utilizing the *rpb1-1 *mutant strain must be treated with caution, as PolII remains associated with the genome for much longer than previously appreciated at the restrictive temperature.

## Materials and methods

### Yeast culture

Two strains were used - the *rpb1-1 *mutant (gift from Fred Winston), and parental strain BY4741. For the diamide time course, five flasks each of 250 ml *rpb1-1 *cells were grown in YPD to an A_600 _OD of 0.5 in 1-l flasks shaking at 200 rpm at room temperature (25°C). Diamide was added to a final concentration of 1.5 mM to flasks at time zero. At t = 0, 15, 30, 60, and 120 minutes, formaldehyde was added to a final concentration of 1%. For the heat shock time courses (both wild type and *rpb1-1*), an equal volume of YPD prewarmed to 49°C was added to flasks, which were immediately transferred to a 37°C incubator and incubated for varying lengths of time prior to fixation. Finally, for heat shock plus diamide treatment, three flasks of *rpb1-1 *cells were shifted to 37°C as above for 10 minutes, then diamide was added for 15, 30, or 60 minutes prior to fixation.

### Chromatin immunoprecipitation and DNA amplification

We carried out PolII ChIP as previously described with minor modifications [16,17]. protein G beads (20 μl) and anti-Rpb3 monoclonal antibody (5 μl) were mixed with 1 ml of chromatin solution containing 1 mg of proteins and incubated overnight at 4°C. Beads were washed sequentially with 1 ml each of FA lysis buffer containing 275 mM NaCl, FA lysis buffer containing 500 mM NaCl, wash buffer (10 mM Tris, pH 8.0, 0.25 M LiCl, 1 mM EDTA, 0.5% NP-40, 0.5% Na deoxycholate), and TE (10 mM Tris pH 8.0, 0.1 mM EDTA). Immunoprecipitated chromatin was eluted from the beads by heating for 20 minutes at 65°C in 200 μl of 50 mM Tris, pH 7.5, 10 mM EDTA, and 1% SDS. After recovery of the supernatant, beads were washed with 200 ml TE that was then added to the first supernatant. For Input DNA, 150 μl of FA lysis buffer and 200 μl of TE were added into 50 μl of chromatin solution. Reversal of crosslinking was done as described [41], and then the precipitated DNA and Input DNA were resolved in 45 μl and 50 μl of distilled water, respectively. Precipitated DNA (40 μl) and 1/50 diluted Input DNA were used for amplification. DNA amplification was done as described previously [17].

### Microarray hybridization

DNA produced from the amplification (3 μg) was used to label probe via Klenow labeling. Labeled probes were hybridized onto a yeast tiled oligonucleotide microarray at 65°C for 16 hours and washed as described [23]. The arrays were scanned at 5 micron resolution with an Axon Laboratories GenePix 4000B scanner running GenePix 5.1 (Molecular Devices, Sunnyvale, CA, USA).

### Data availability

Data have been deposited to the Gene Expression Omnibus, accession number [GEO:GSE22675].

## Abbreviations

bp: base pair; CDS: coding sequence; ChIP: chromatin immunoprecipitation; CUT: cryptic unstable transcript; ORF: open reading frame; PolII: RNA polymerase II; SUT: stable unannotated transcript.

## Authors' contributions

TSK designed experiments and carried out Pol2 ChIPs and amplifications, CLL designed experiments, JH performed microarray hybridizations, MY assisted with data analysis, NF participated in experiment planning and manuscript preparation, SB participated in experiment planning and manuscript preparation, and OJR conceived of the study, performed data analysis, and drafted the manuscript. All authors read and approved the final manuscript.

## Supplementary Material

Additional file 1 **Table S1**. Complete dataset. PolII localization dataset for 18 experiments, as indicated in the column headings. Microarray probes are identified both by Agilent probe ID as well as by chromosome coordinate. Click here for file

Additional file 2 **Table S2**. Granularized data. Probes were grouped as described in [16], and data were averaged for varying annotations (that is, YAL001W-5CDS indicates probes falling within 500 bp of YAL001W's ATG) as indicated. Click here for file

Additional file 3 **Table S3**. PolII localization sorted by 5'/3' bias. For genes with a mid-CDS annotation (that is, genes over 1 kb in length), data are shown for 5' CDS, mid-CDS, and 3' CDS. Genes are ordered by the ratio of 5' to 3' PolII abundance. Click here for file

Additional file 4 **Figure S1**. Genes with 3'-biased PolII are poorly-expressed. **(a) **Genes are sorted by 5' bias in PolII abundance as in Figure 2b, but all genes with overlapping CUTs and SUTs have been removed. **(b) **Genes with high 5'/3' PolII abundance are highly expressed. Log(2) of mRNA abundance data from Yassour *et al*. [21] is shown as an 80-gene running-window average, with genes ordered as in (a). Click here for file

Additional file 5 **Figure S2**. Comparison of PolII location on gene body to mRNA induction level. **(a, b) **PolII enrichment was calculated for 5', mid-, and 3' CDS at t = 0 (pre-stress) and t = 30 minutes (stress) for heat shock (a), and diamide (b) stresses. Genes are ordered by mRNA change from highly repressed (left) to highly activated (right), and PolII abundances are plotted as an 80-gene running-window average. Genes that are highly repressed (left) tend to exhibit 5'-biased PolII pre-stress. Click here for file

Additional file 6 **Figure S3**. Comparison of PolII changes to mRNA changes during the diamide stress response. **(a) **mRNA data from Gasch *et al*. [18] were subjected to k-means clustering with k = 8. **(b, c) **PolII data for 5' (b) or 3' (c) CDS were normalized by subtracting t = 0 data from each time point, and genes long enough to have a mid-CDS annotation are ordered as in (a). **(d) **Average mRNA changes for the eight clusters from (a), with cluster number indicated by the color to the right of the data in (a). **(e) **As in (d), but for mid-CDS PolII occupancy changes. Click here for file

Additional file 7 **Figure S4**. Comparison of PolII changes to mRNA changes during two stress responses. **(a-f) **mRNA data from Gasch *et al*. [18] are plotted on the y-axis, with the change in PolII at mid-CDS for the same gene plotted on the x-axis. The red line shows an 80-gene running-window average. HS refers to heat shock, D refers to diamide, and 15, 30, and 60 refer to minutes of stress. Note for diamide the mRNA data come from 20 rather than 15 minutes. Click here for file

Additional file 8 **Figure S5**. Genes involved in carbohydrate metabolism exhibit significant excess mRNA produced per PolII change. **(a) **Scatterplot as in Figure 4a,c and Additional file 7. Red triangles indicate genes annotated with 'carbohydrate metabolic process'. **(b) **Cumulative distribution plots for carbohydrate metabolism genes (red) and all others (blue) showing that a significantly higher fraction of carbohydrate metabolism genes exhibit excess mRNA production relative to the background distribution. Click here for file

Additional file 9 **Figure S6**. Genes producing 'excess' mRNA exhibit significant overlap with CUTs and SUTs. Cumulative distribution (y-axis) of genes overlapping a given length of alternative transcript [22], summed over both 5' and 3' overlaps, for genes exhibiting excess (red), predicted (blue), or a dearth of (green) mRNA production per PolII change (Figure 4c). Click here for file

Additional file 10 **Figure S7**. Model for excess mRNA production per PolII. Before stress, a gene with an overlapping CUT will be associated with PolII molecules producing mRNA (right arrow) as well as PolII molecules producing rapidly degraded 'cryptic' transcripts (left arrows). After stress, repression of the CUT promoter and activation of the ORF promoter will result in a greater proportion of mRNA-producing PolII molecules associated with the ORF. Note that either ORF promoter activation or CUT promoter repression alone would be sufficient to increase the relative proportion of productive PolII relative to overall PolII, but both are shown here to illustrate the point. Click here for file

Additional file 11 **Figure S8**. Genes with high 3'/5' PolII ratios pre-stress tend to produce excess mRNA during stress. **(a) **Scatterplot of PolII 5'/3' ratio at t = 0 (as in Figure 2b), x axis, versus mRNA excess produced after 30 minutes of heat shock. **(b) **Eighty-gene running-window average of the data from (a). Note the y-axis scale changes between these two panels. Click here for file

Additional file 12 **Figure S9**. mRNA data collected in this study correlate with Gasch *et al*. [18]. **(a) **Comparison of mRNA changes from Gasch *et al*. [18] with those measured in this study after 30 minutes of heat shock. Axes are log(2) of the fold-change relative to t = 0. **(b) **mRNA changes compared to PolII changes over mid-CDS. As in Figure 4c, but for mRNA data collected in this study. **(c) **Genes exhibiting 'excess' mRNA production per PolII more extensively overlap CUTs and SUTs than do other genes. As in Figure 4d. Click here for file

Additional file 13 **Figure S10**. PolII abundance before and after inactivation of *rpb1-1*. **(a-c) **Scatterplots of PolII abundance before stress (a), after 120 minutes of heat shock in wild type (b), or after 30 minutes of inactivation of *rpb1-1 *(c), on the x-axis, versus PolII abundance after 120 minutes of heat shock in *rpb1-1 *(y-axis). Running window averages are indicated for 5', mid-, and 3' CDS probes. Click here for file

Additional file 14 **Figure S11**. Excess mRNA production correlates with mRNA half-life. Genes are ordered according to deviation from expected mRNA produced per change in PolII (Figure 4c) for 30 minutes of heat shock, and an 80-gene running-window average of mRNA half-life [2] is plotted on the y-axis. Click here for file
