# Supplementary figures and images for: RNA polymerase mapping during stress responses reveals widespread nonproductive transcription in yeast
Source: Genome Biol. 2010 Jul 16;11(7):R75. doi: 10.1186/gb-2010-11-7-r75 (PMC2926786; doi:10.1186/gb-2010-11-7-r75)

Figure S1

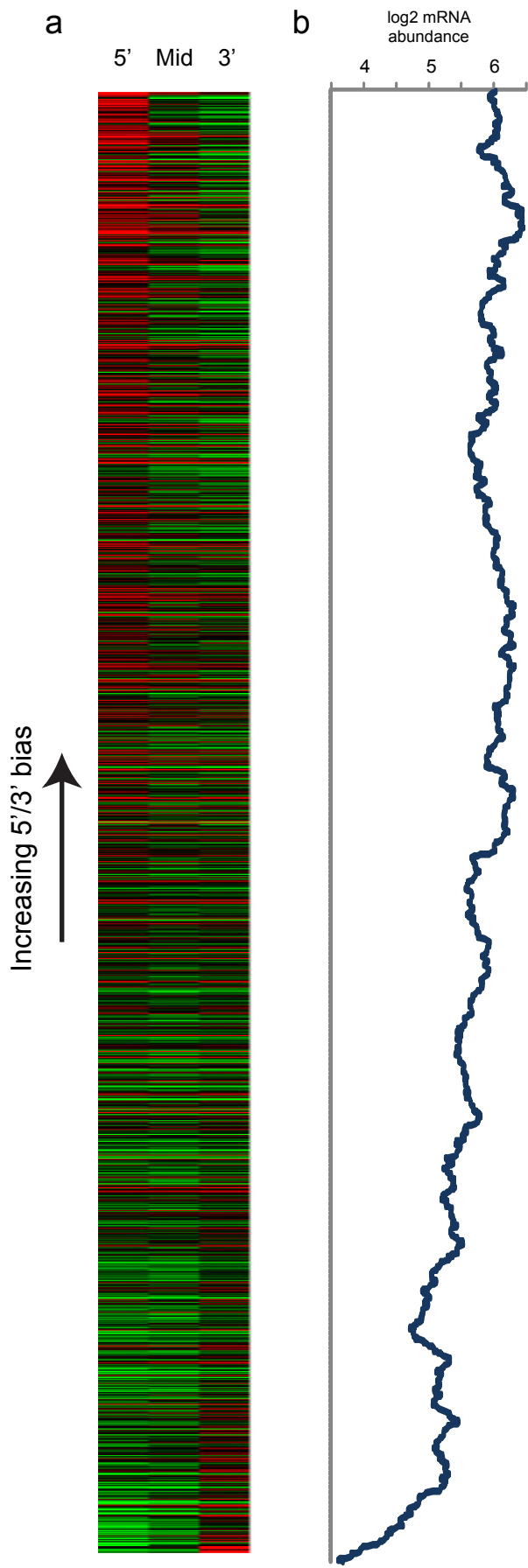

Supplement: Additional file 4 — Figure S1. Genes with 3'-biased PolII are poorly-expressed. (a) Genes are sorted by 5' bias in PolII abundance as in Figure 2b, but all genes with overlapping CUTs and SUTs have been removed. (b) Genes with high 5'/3' PolII abundance are highly expressed. Log(2) of mRNA abundance data from Yassour et al. [21] is shown as an 80-gene running-window average, with genes ordered as in (a). [file gb-2010-11-7-r75-S4.PDF]

Figure S2

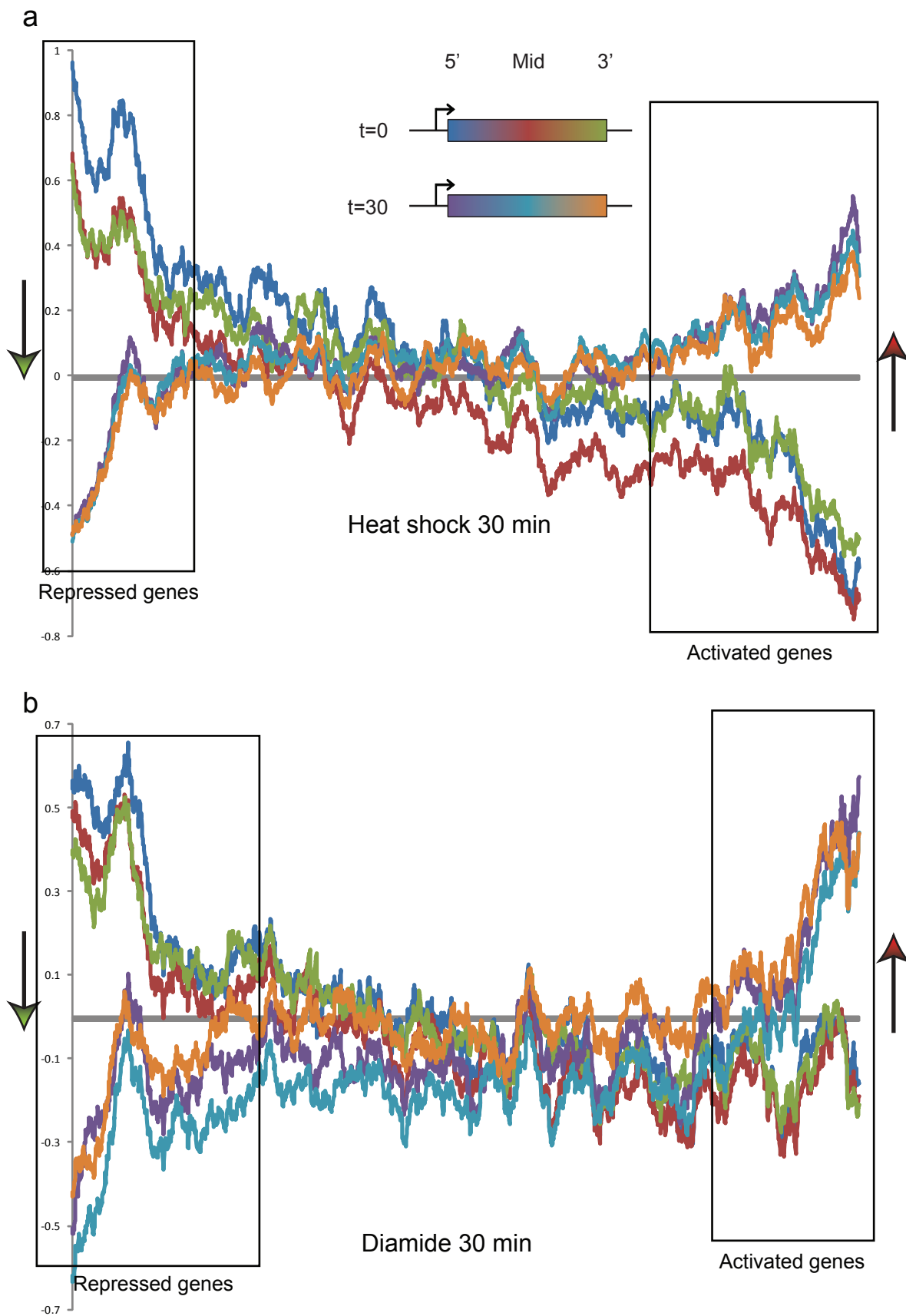

Supplement: Additional file 5 — Figure S2. Comparison of PolII location on gene body to mRNA induction level. (a, b) PolII enrichment was calculated for 5', mid-, and 3' CDS at t = 0 (pre-stress) and t = 30 minutes (stress) for heat shock (a), and diamide (b) stresses. Genes are ordered by mRNA change from highly repressed (left) to highly activated (right), and PolII abundances are plotted as an 80-gene running-window average. Genes that are highly repressed (left) tend to exhibit 5'-biased PolII pre-stress. [file gb-2010-11-7-r75-S5.PDF]

Figure S3

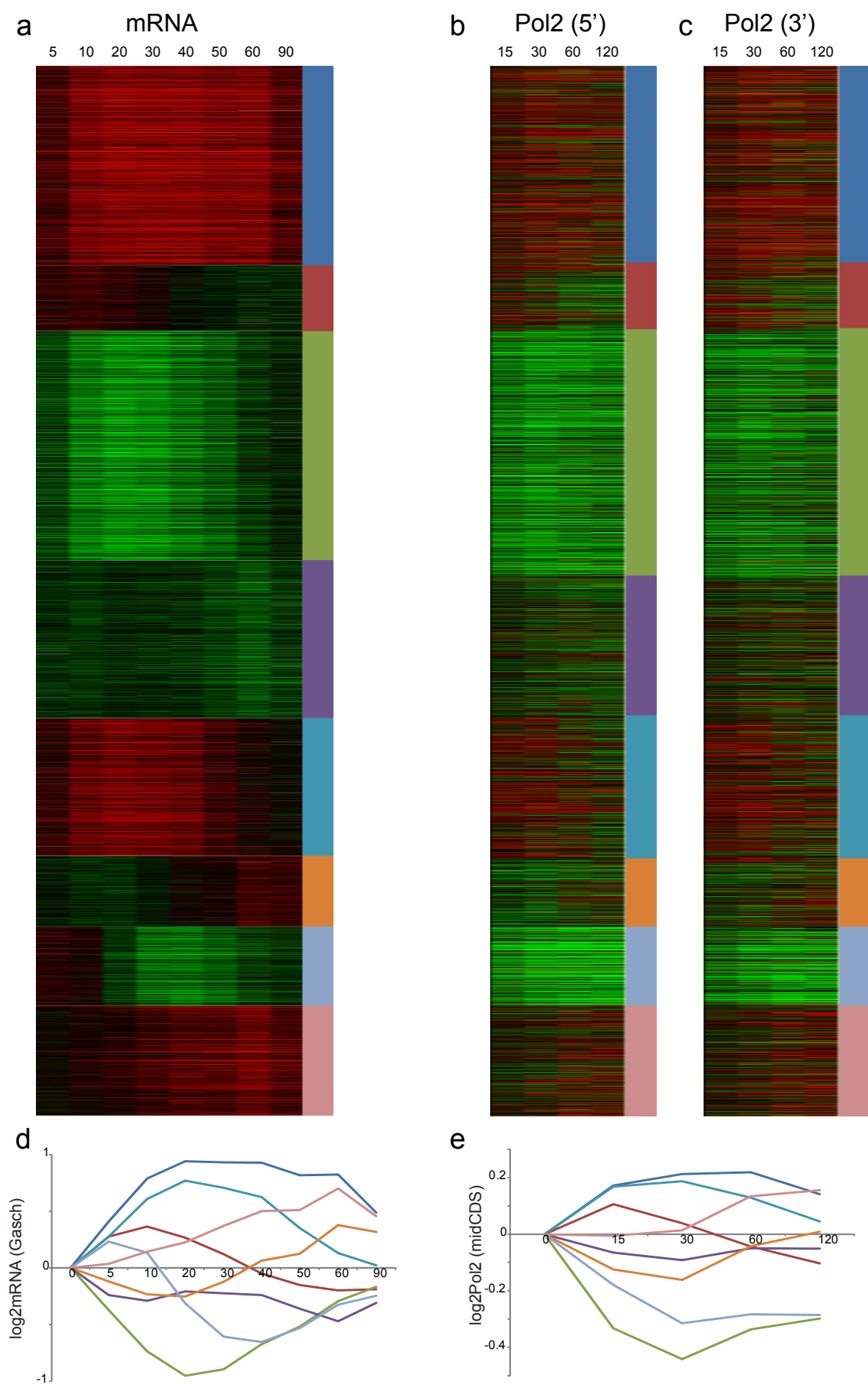

Supplement: Additional file 6 — Figure S3. Comparison of PolII changes to mRNA changes during the diamide stress response. (a) mRNA data from Gasch et al. [18] were subjected to k-means clustering with k = 8. (b, c) PolII data for 5' (b) or 3' (c) CDS were normalized by subtracting t = 0 data from each time point, and genes long enough to have a mid-CDS annotation are ordered as in (a). (d) Average mRNA changes for the eight clusters from (a), with cluster number indicated by the color to the right of the data in (a). (e) As in (d), but for mid-CDS PolII occupancy changes. [file gb-2010-11-7-r75-S6.PDF]

Figure S4

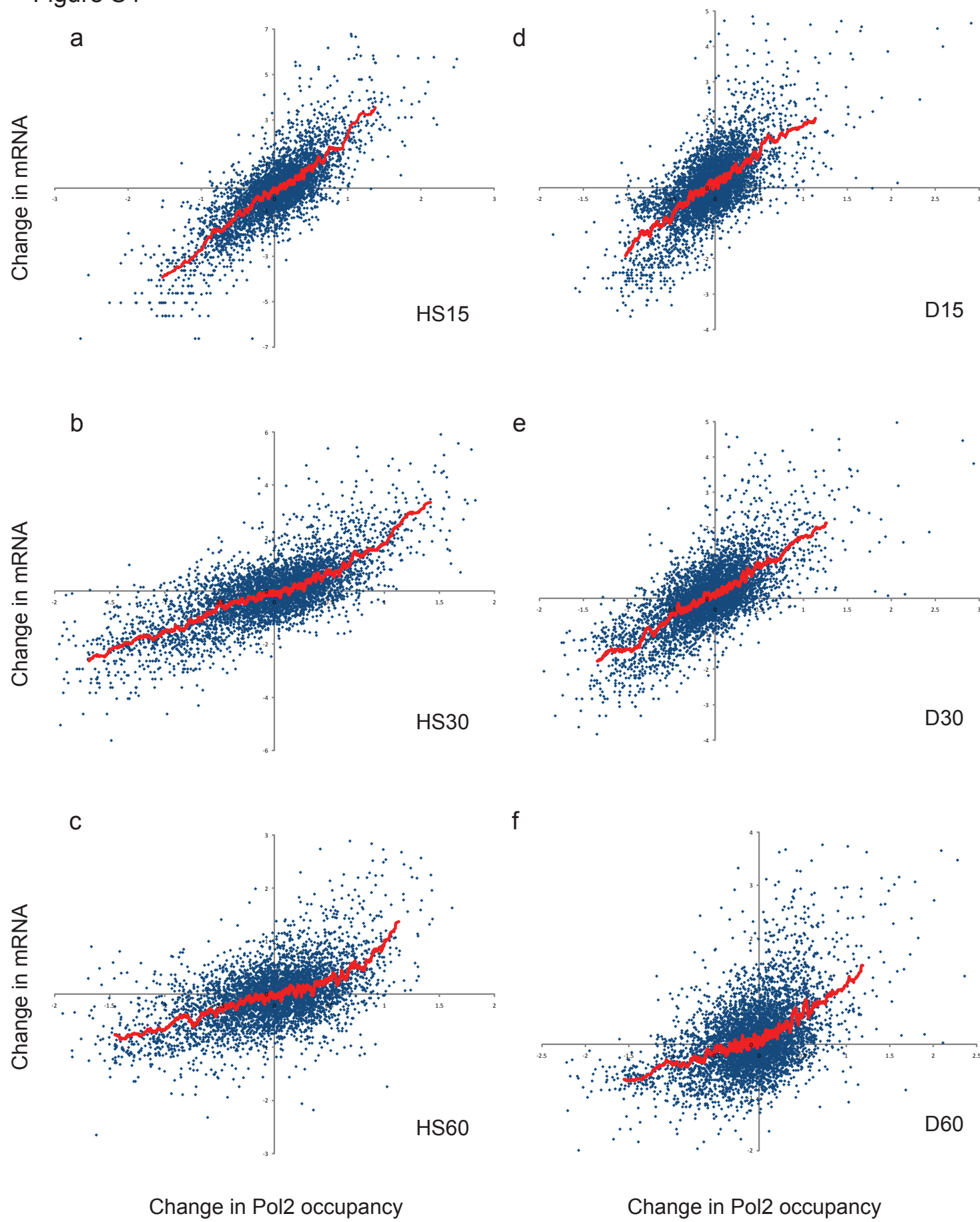

Supplement: Additional file 7 — Figure S4. Comparison of PolII changes to mRNA changes during two stress responses. (a-f) mRNA data from Gasch et al. [18] are plotted on the y-axis, with the change in PolII at mid-CDS for the same gene plotted on the x-axis. The red line shows an 80-gene running-window average. HS refers to heat shock, D refers to diamide, and 15, 30, and 60 refer to minutes of stress. Note for diamide the mRNA data come from 20 rather than 15 minutes. [file gb-2010-11-7-r75-S7.PDF]

Figure S5

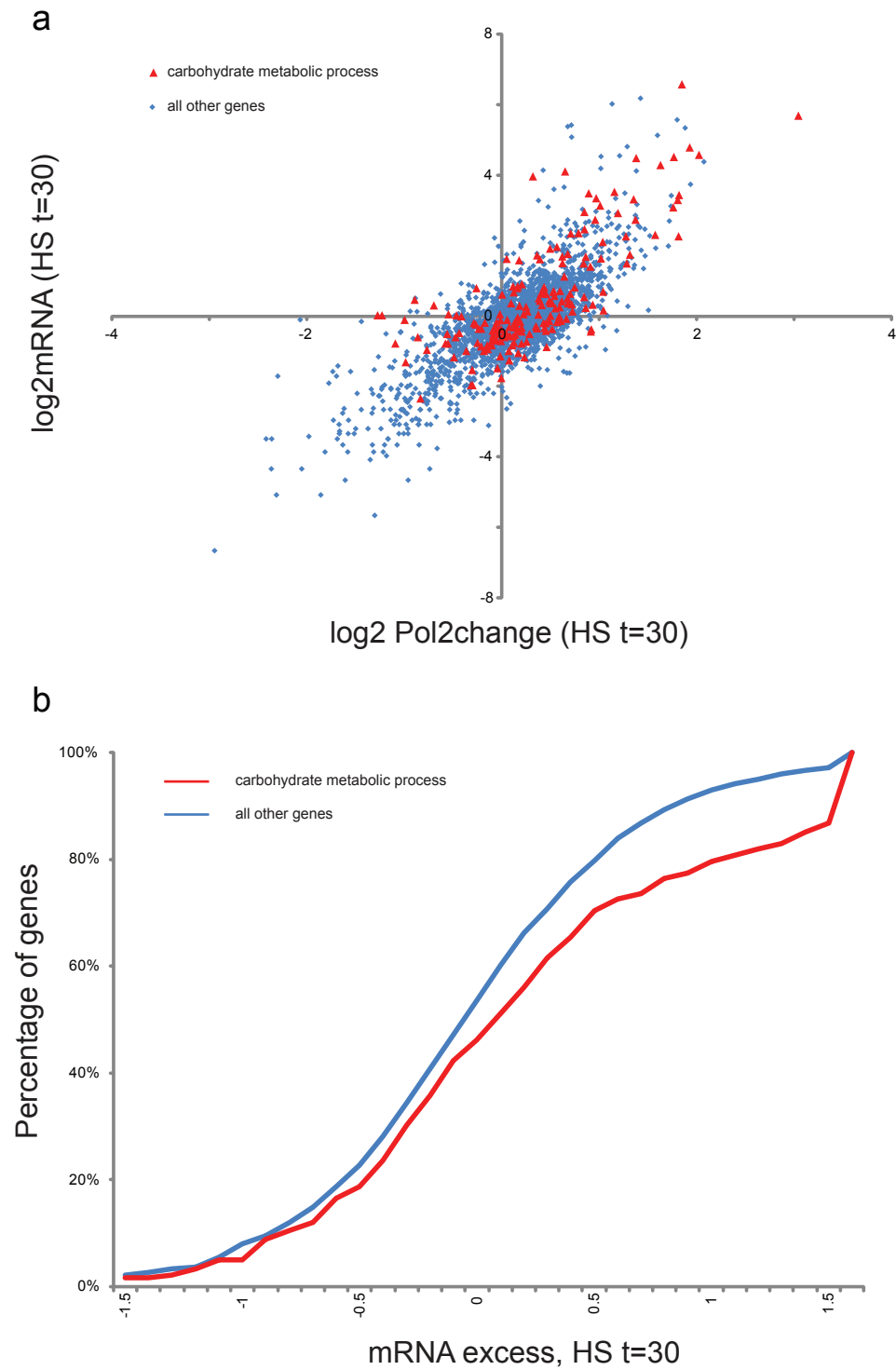

Supplement: Additional file 8 — Figure S5. Genes involved in carbohydrate metabolism exhibit significant excess mRNA produced per PolII change. (a) Scatterplot as in Figure 4a,c and Additional file 7. Red triangles indicate genes annotated with 'carbohydrate metabolic process'. (b) Cumulative distribution plots for carbohydrate metabolism genes (red) and all others (blue) showing that a significantly higher fraction of carbohydrate metabolism genes exhibit excess mRNA production relative to the background distribution. [file gb-2010-11-7-r75-S8.PDF]

Figure S6

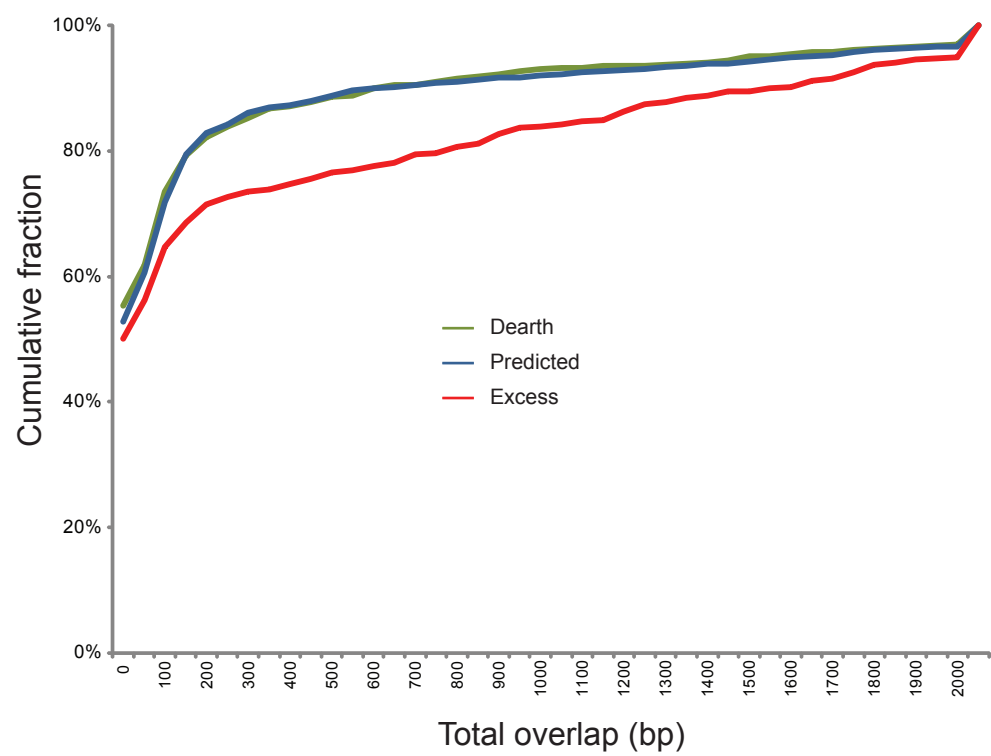

Supplement: Additional file 9 — Figure S6. Genes producing 'excess' mRNA exhibit significant overlap with CUTs and SUTs. Cumulative distribution (y-axis) of genes overlapping a given length of alternative transcript [22], summed over both 5' and 3' overlaps, for genes exhibiting excess (red), predicted (blue), or a dearth of (green) mRNA production per PolII change (Figure 4c). [file gb-2010-11-7-r75-S9.PDF]

Figure S7

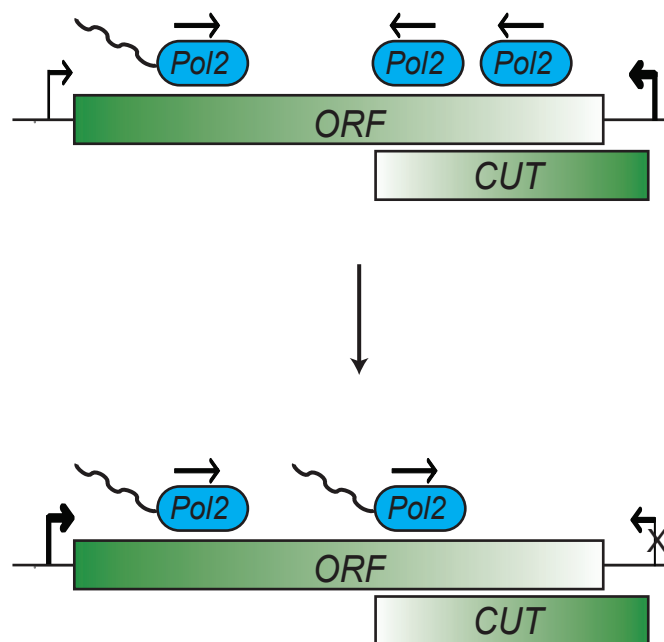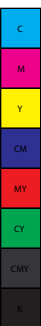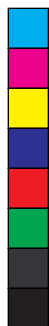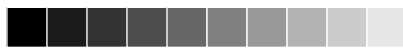

Supplement: Additional file 10 — Figure S7. Model for excess mRNA production per PolII. Before stress, a gene with an overlapping CUT will be associated with PolII molecules producing mRNA (right arrow) as well as PolII molecules producing rapidly degraded 'cryptic' transcripts (left arrows). After stress, repression of the CUT promoter and activation of the ORF promoter will result in a greater proportion of mRNA-producing PolII molecules associated with the ORF. Note that either ORF promoter activation or CUT promoter repression alone would be sufficient to increase the relative proportion of productive PolII relative to overall PolII, but both are shown here to illustrate the point. [file gb-2010-11-7-r75-S10.PDF]

Figure S8

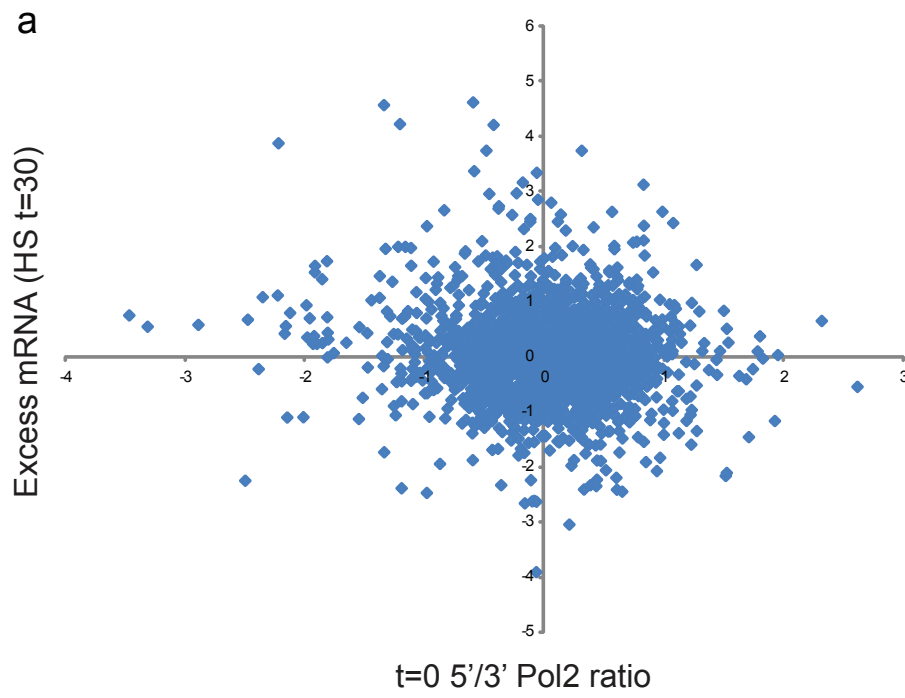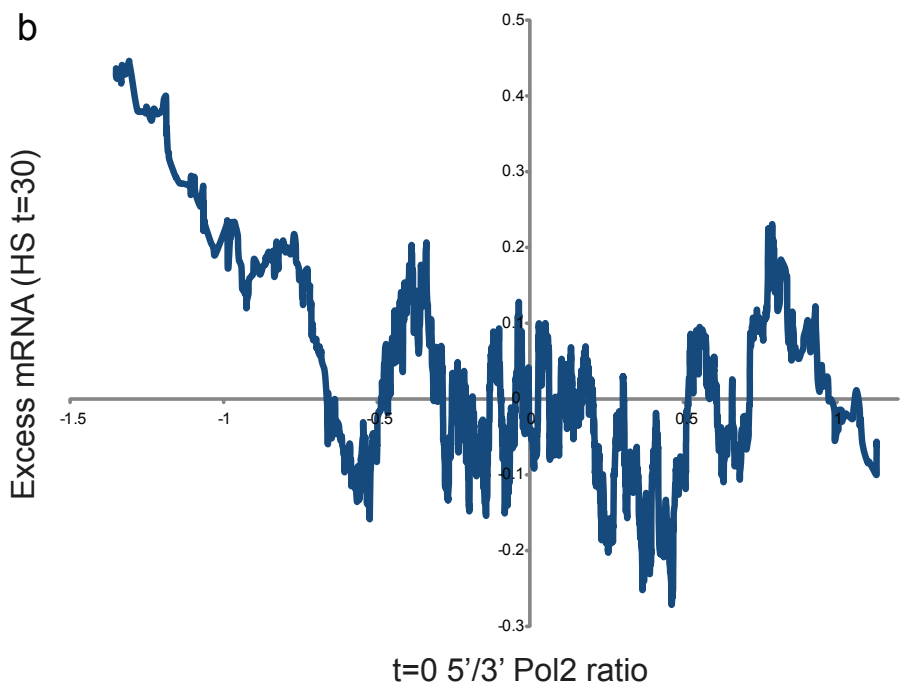

Supplement: Additional file 11 — Figure S8. Genes with high 3'/5' PolII ratios pre-stress tend to produce excess mRNA during stress. (a) Scatterplot of PolII 5'/3' ratio at t = 0 (as in Figure 2b), x axis, versus mRNA excess produced after 30 minutes of heat shock. (b) Eighty-gene running-window average of the data from (a). Note the y-axis scale changes between these two panels. [file gb-2010-11-7-r75-S11.PDF]

Figure S9

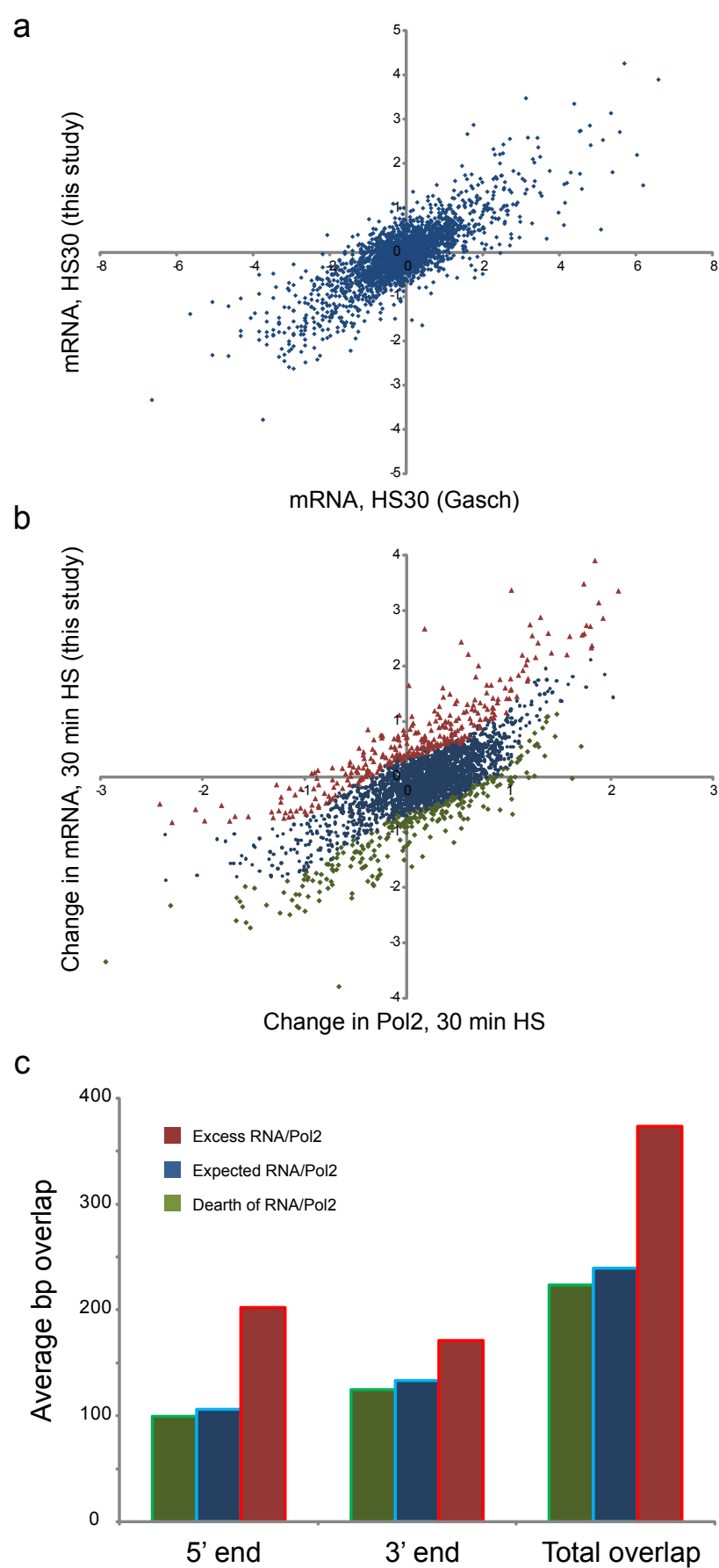

Supplement: Additional file 12 — Figure S9. mRNA data collected in this study correlate with Gasch et al. [18]. (a) Comparison of mRNA changes from Gasch et al. [18] with those measured in this study after 30 minutes of heat shock. Axes are log(2) of the fold-change relative to t = 0. (b) mRNA changes compared to PolII changes over mid-CDS. As in Figure 4c, but for mRNA data collected in this study. (c) Genes exhibiting 'excess' mRNA production per PolII more extensively overlap CUTs and SUTs than do other genes. As in Figure 4d. [file gb-2010-11-7-r75-S12.PDF]

Figure S10

a

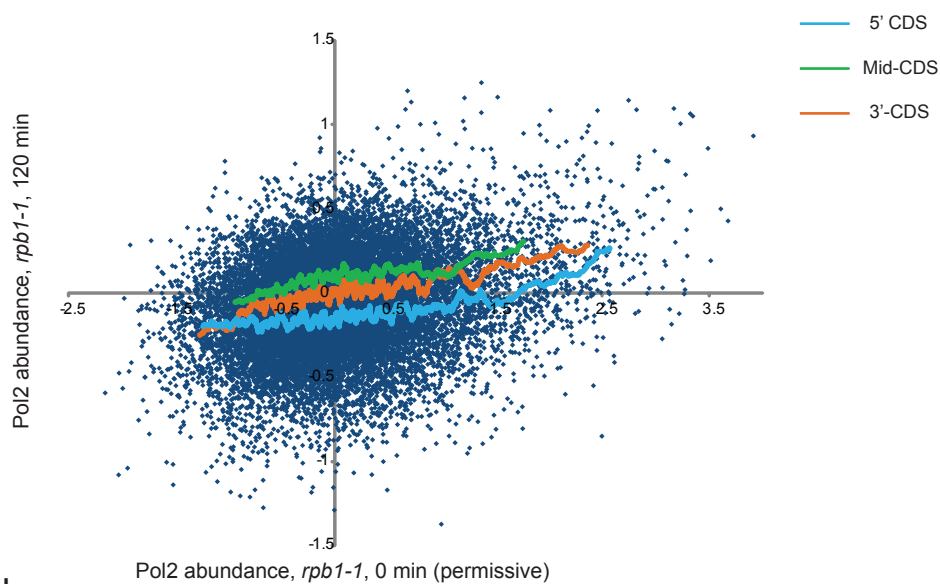

b

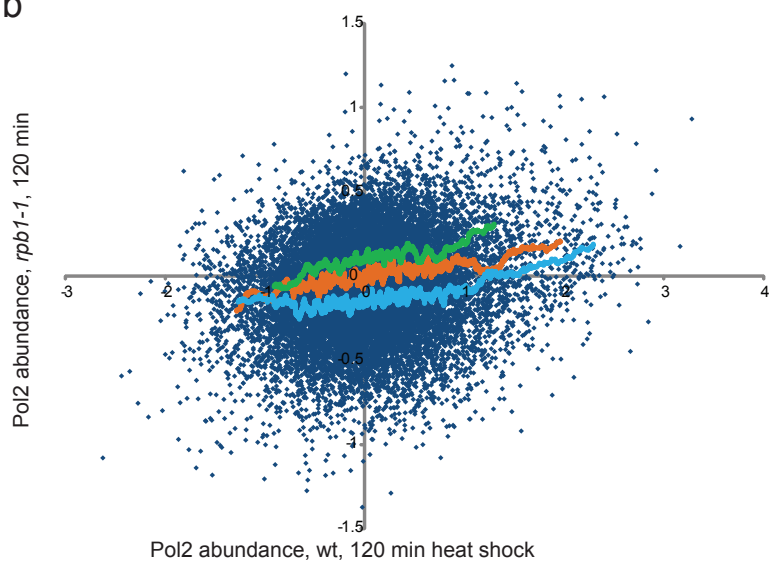

c

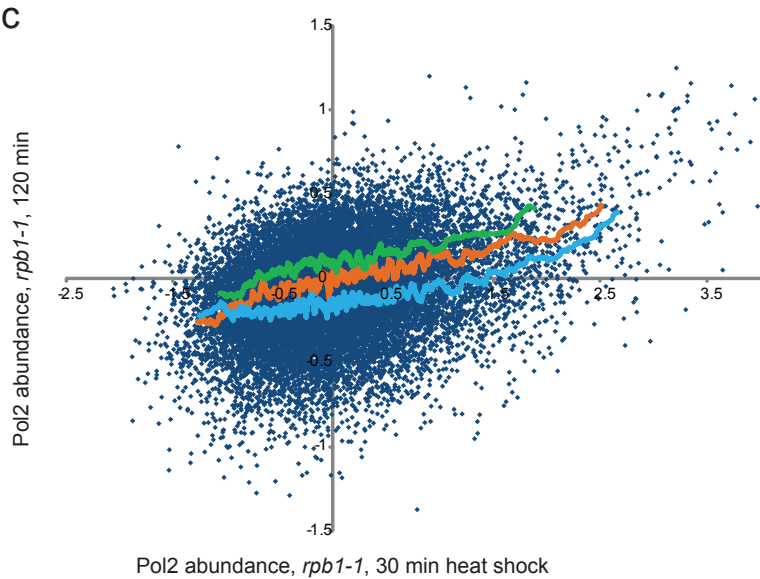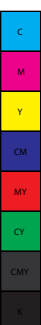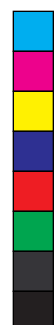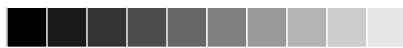

Supplement: Additional file 13 — Figure S10. PolII abundance before and after inactivation of rpb1-1. (a-c) Scatterplots of PolII abundance before stress (a), after 120 minutes of heat shock in wild type (b), or after 30 minutes of inactivation of rpb1-1 (c), on the x-axis, versus PolII abundance after 120 minutes of heat shock in rpb1-1 (y-axis). Running window averages are indicated for 5', mid-, and 3' CDS probes. [file gb-2010-11-7-r75-S13.PDF]

Figure S11

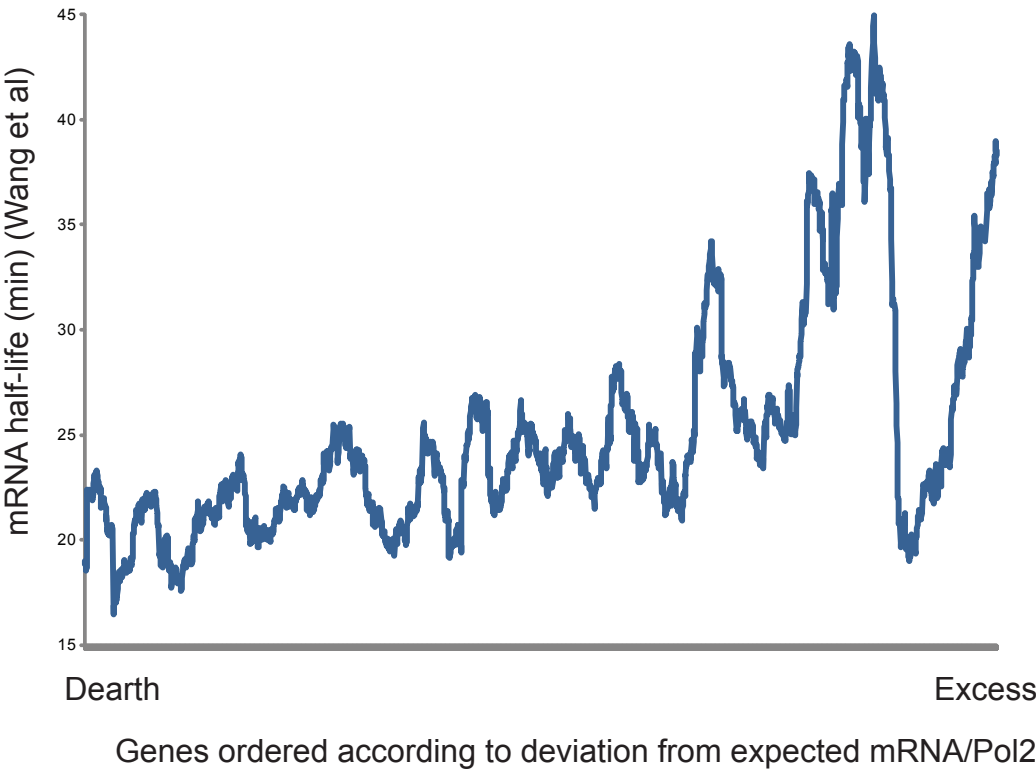

Supplement: Additional file 14 — Figure S11. Excess mRNA production correlates with mRNA half-life. Genes are ordered according to deviation from expected mRNA produced per change in PolII (Figure 4c) for 30 minutes of heat shock, and an 80-gene running-window average of mRNA half-life [2] is plotted on the y-axis. [file gb-2010-11-7-r75-S14.PDF]
